# Computational Analysis of HIV-1 Resistance Based on Gene Expression Profiles and the Virus-Host Interaction Network

**DOI:** 10.1371/journal.pone.0017291

**Published:** 2011-03-04

**Authors:** Tao Huang, Zhongping Xu, Lei Chen, Yu-Dong Cai, Xiangyin Kong

**Affiliations:** 1 Key Laboratory of Systems Biology, Shanghai Institutes for Biological Sciences, Chinese Academy of Sciences, Shanghai, People's Republic of China; 2 Shanghai Center for Bioinformation Technology, Shanghai, People's Republic of China; 3 State Key Laboratory of Medical Genomics, Shanghai Institute of Hematology, Shanghai Jiao Tong University School of Medicine, RuiJin Hospital, Shanghai, People's Republic of China; 4 Centre for Computational Systems Biology, Fudan University, Shanghai, People's Republic of China; 5 College of Information Engineering, Shanghai Maritime University, Shanghai, People's Republic of China; 6 Institute of Systems Biology, Shanghai University, Shanghai, People's Republic of China; 7 Key Laboratory of Stem Cell Biology, Institute of Health Sciences, Shanghai Institutes for Biological Sciences, Chinese Academy of Sciences and Shanghai Jiao Tong University School of Medicine, Shanghai, People's Republic of China; Massachusetts General Hospital, United States of America

## Abstract

A very small proportion of people remain negative for HIV infection after repeated HIV-1 viral exposure, which is called HIV-1 resistance. Understanding the mechanism of HIV-1 resistance is important for the development of HIV-1 vaccines and Acquired Immune Deficiency Syndrome (AIDS) therapies. In this study, we analyzed the gene expression profiles of CD4+ T cells from HIV-1-resistant individuals and HIV-susceptible individuals. One hundred eighty-five discriminative HIV-1 resistance genes were identified using the Minimum Redundancy-Maximum Relevance (mRMR) and Incremental Feature Selection (IFS) methods. The virus protein target enrichment analysis of the 185 HIV-1 resistance genes suggested that the HIV-1 protein nef might play an important role in HIV-1 infection. Moreover, we identified 29 infection information exchanger genes from the 185 HIV-1 resistance genes based on a virus-host interaction network analysis. The infection information exchanger genes are located on the shortest paths between virus-targeted proteins and are important for the coordination of virus infection. These proteins may be useful targets for AIDS prevention or therapy, as intervention in these pathways could disrupt communication with virus-targeted proteins and HIV-1 infection.

## Introduction

Acquired immunodeficiency syndrome (AIDS) is caused by human immunodeficiency virus (HIV), which is a member of the retrovirus family [1]. According to UNAIDS Outlook 2010 [2], there were 33.4 million people infected with HIV in July 2010, 2 million deaths per year, and 2.7 million new infections per year. HIV destroys the human immune system through infection of helper T cells (CD4+ T cells), macrophages, and dendritic cells [3].

Infection with HIV-1 does not always lead to AIDS [4] because different people have different responses to HIV-1 infection. Very small proportions of individuals are resistant to HIV-1 infection and remain negative after repeated HIV-1 viral exposure [5], [6], [7]. The mechanism of HIV-1 resistance in these individuals could be used to design an HIV-1 vaccine, which is crucial for containing the spread of HIV. Microarray technology makes it possible to measure the expression of thousands of genes. Information on protein interactions can help us understand the mechanisms of biological problems [8], [9], [10]. The combination of these technologies may allow us to elucidate the mechanisms of HIV-1 infection and resistance.

In this study, we analyzed a published dataset that included 85 samples from HIV-1-resistant individuals and 50 samples from HIV low-risk negative individuals [11]. The gene expression profiles of CD4+ T cells were measured using the NIA/NIH Human Focused Immune Array 4600. One hundred eight-five discriminative genes were identified with the Minimum Redundancy-Maximum Relevance (mRMR) principle and Incremental Feature Selection (IFS) method. The prediction accuracy of the 185-gene signature using the Nearest Neighbor Algorithm (NNA) was 85.2% according to Leave-One-Out Cross-Validation (LOOCV). To interpret the relevance of the 185 genes to HIV-1 resistance, we investigated the virus-host protein interaction network, which integrated the HIV-1, human protein interaction database [12] and the STRING database [13]. We found that the 185 genes were enriched in targets of the HIV-1 protein nef, which suggests that nef plays an important role in HIV-1 infection. In addition, we identified 29 genes from the 185 genes that may disrupt the communication between virus-targeted proteins based on the network analysis. These genes are located on the shortest paths between virus-targeted proteins and are important for exchanging information between virus-targeted proteins and coordinating virus invasion. Targeting of these genes may disrupt the communication between virus-targeted proteins and infection, and they may serve as novel drug targets for Acquired Immune Deficiency Syndrome (AIDS) therapy or prevention.

## Methods

### Microarray Dataset

The microarray data used in this work were from Paul J. McLaren's study [11] of HIV-1-resistant individuals and HIV-1-susceptible individuals. Their data are publicly available at GEO (http://www.ncbi.nlm.nih.gov/geo/query/acc.cgi?acc=GSE14279). There were 85 samples from HIV-1-resistant individuals and 50 samples from HIV low-risk negative individuals. The NIA/NIH Human Focused Immune Array 4600 was used to measure the gene expression profiles of CD4+ T cells from those samples. After averaging the duplicate probes for genes and quantile normalization, we obtained the expression profiles of 1868 genes in 85 HIV-1-resistant samples and 50 HIV-1-susceptible samples.

### Minimum Redundancy-Maximum Relevance (mRMR) feature selection

As a widely used feature selection method, Minimum Redundancy-Maximum Relevance (mRMR) [14] is designed to select features that best classify the target variable. The selected features by mRMR are as similar as possible to the classification variable and as dissimilar as possible to each other. The mRMR program can be downloaded from http://penglab.janelia.org/proj/mRMR/. The mRMR program gives two scores: the score in the MaxRel list is the relevance score between the features and the target; the score in the mRMR list maximizes the relevance and minimizes the redundancy. In our study, we used the mRMR list.

### Classifier Construction and Evaluation

In this study, we used the Nearest Neighbor Algorithm (NNA; available from http://pcal.biosino.org/NNA.html) [15] to classify the samples into the HIV-1-resistant and HIV-1-susceptible groups based on the cosine similarity [16], [17], [18] between the query patient and each of the patients in the training set. The query patient is predicted to be in the same group as that of its nearest neighbor in the training set.

To evaluate the constructed prediction model, Leave-One-Out Cross-Validation (LOOCV) [16], [17], [18], [19], [20] was applied. During LOOCV, each sample in the dataset was used as a test sample and predicted based on the model trained with the other samples. The prediction accuracy was used to evaluate the prediction performance:
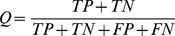
(1)where TP, TN, FP and FN stand for the numbers of true positive, true negative, false positive and false negative samples, respectively.

### Incremental Feature Selection (IFS)

mRMR only sorts features according to their importance, but whether more features should be selected was still not known. In this study, Incremental Feature Selection (IFS) [16], [17], [18], [20] was used to determine the optimal number of features. By testing all of the possible top feature sets, the feature set that produces the highest prediction accuracy is chosen as the optimal feature set. The possible feature subset 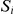 can be expressed as:
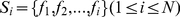
(2)where N is the total number of features. The leave-one-out test was used to obtain the prediction accuracies of the different feature sets. The feature set that produced the highest prediction accuracy is the optimal feature set. To visualize the IFS process, we plotted an IFS curve in which the x-axis is the number of features and the y-axis is the prediction accuracies.

### Communication between virus-targeted proteins in a weighted interaction network

To investigate the virus-host interaction, we downloaded the HIV-1, human protein interaction network from the National Institute of Allergy and Infectious Diseases (http://www.ncbi.nlm.nih.gov/RefSeq/HIVInteractions/) [12] and the human protein interaction network from STRING (http://string.embl.de/) [13], which is a large database of known and predicted protein interactions. Because the HIV-1, human protein interaction database used Entrez Gene IDs and STRING used Ensembl Peptide IDs, we transformed the Entrez Gene IDs in the HIV-1, human protein interaction database into Ensembl Peptide IDs and Gene Symbols using BioMart [21]. The ID transformed HIV-1, human protein interactions are available in **Table S1**.

Dijkstra's algorithm [22] was applied to obtain the shortest paths between virus-targeted human proteins in the weighted interaction network. The weight in the protein interaction network was defined as one minus the confidence score in STRING v8.3. Inspired by Freeman's betweenness [23], which measures the information flow through a network, we calculated the modified betweenness of node 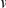 in graph 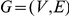:
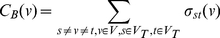
(3)where 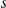 and 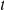 are nodes from the virus-targeted proteins 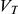 in the network, and 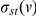 is whether the shortest path between nodes 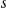 and 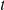 goes through node 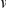. A node with high modified betweenness may be important for the exchange of information between virus-targeted proteins and may be targets for the disruption of communication between virus-targeted proteins and the prevention of virus infection.

### The analysis workflow of HIV-1 resistance genes

**Figure 1:**
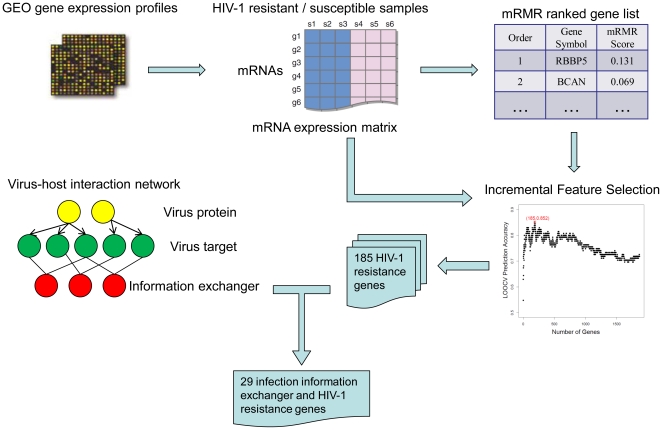
Workflow of the HIV-1 resistance gene analysis. First, we used mRMR to rank the genes based on their relevance to HIV-1 resistance. Second, IFS was applied to optimize the HIV-1 resistance prediction model and identify 185 optimal HIV-1 resistance genes. Then, we obtained the information exchanger genes based on the virus-host interaction network and compared them with the HIV-1 resistance genes indentified by mRMR and IFS. Finally, we identified 29 infection information exchanger and HIV-1 resistance genes.

Our strategy for the analysis of HIV-1 resistance genes is demonstrated in **Figure 1**. First, we used mRMR to rank the genes based on their relevance to HIV-1 resistance. Second, IFS was applied to optimize the HIV-1 resistance prediction model and identify the 185 optimal HIV-1 resistance genes. Then, we obtained the information exchanger genes on the virus-host interaction network and compared them with the HIV-1 resistance genes indentified by mRMR and IFS. Finally, we obtained 29 infection information exchanger and HIV-1 resistance genes.

## Results

### Identifying the HIV-1 resistance genes

**Figure 2:**
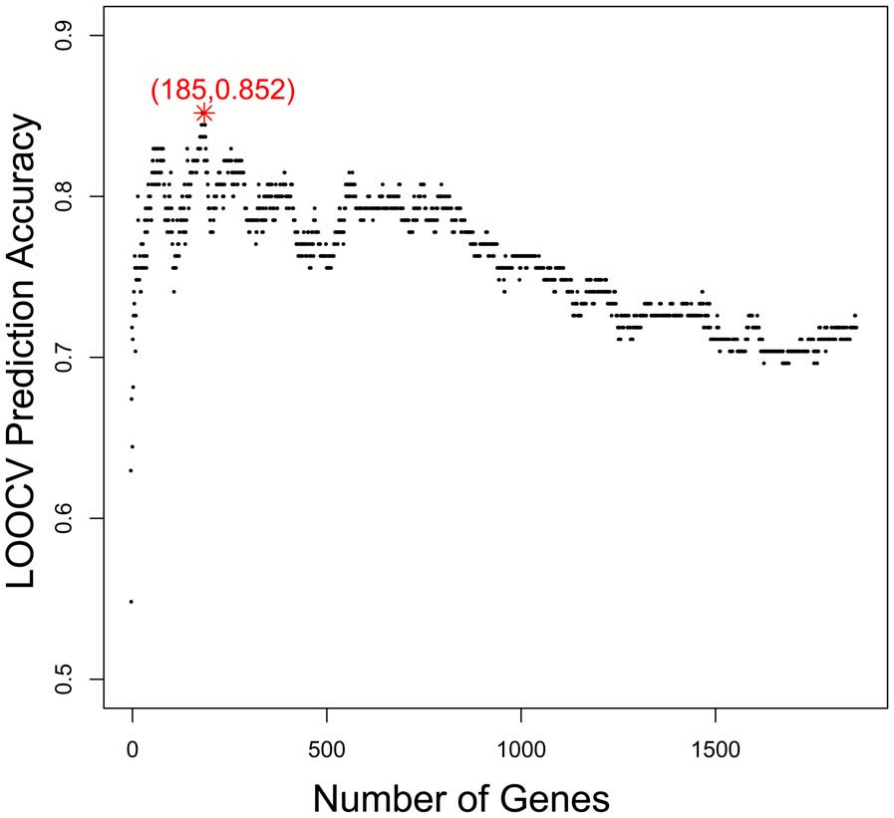
The IFS curve for HIV-1-resistant and susceptible sample classification. In the IFS curve, the x-axis is the number of genes used for classification, and the y-axis is the prediction accuracies of nearest neighbor algorithm evaluated by Leave-One-Out Cross-Validation (LOOCV). The peak accuracy was 0.852 with 185 genes. The top 185 genes in the mRMR gene list formed the optimal discriminative gene set.

To identify HIV-1 resistance genes, we first applied the mRMR method to the expression profiles of 1868 genes in 85 HIV-1-resistant samples and 50 HIV-1-susceptible samples. Then, the 1868 genes were ranked by mRMR according to their importance for discrimination. After the mRMR ranked gene list was obtained, we used the Incremental Feature Selection (IFS) method to determine the optimal discriminative gene set. **Figure 2** shows the IFS curve for optimal gene set selection. The top 185 genes in mRMR gene list formed the optimal discriminative gene set, and the prediction accuracy of this set using the nearest neighbor algorithm was 85.2% according to Leave-One-Out Cross-Validation (LOOCV). **Table S2** shows the complete list of the 185 HIV-1 resistance genes. The code used for HIV-1 resistance gene identification is available by request.

### The biological functions of the 185 HIV-1 resistance genes

To investigate the functions of the 185 HIV-1 resistance genes and their relevance to HIV invasion, we performed Gene Ontology (GO) enrichment and HIV protein targets analyses. **Table S3** shows the Gene Ontology enrichment results for the 185 genes using GATHER [24] (http://gather.genome.duke.edu/) with an adjusted p value smaller than 0.01. These 185 genes were significantly enriched in the response to stress, defense response, immune response, cell communication and signal transduction categories. The Gene Ontology enrichment results were consistent with a previous report that immune responses contributed to protection against HIV-1 infection [25].

### The relationship between the 185 HIV-1 resistance genes and HIV-1 proteins

There are nine HIV-1 proteins: env, gag, nef, pol, rev, tat, vif, vpr and vpu [12]. We wanted to determine which HIV proteins are crucial for HIV invasion or are associated with HIV-1 resistance. We performed a virus target enrichment of the 185 genes using the hypergeometric test [26]. The virus target gene sets were defined as the human protein targets of each HIV-1 protein according to the HIV-1, human protein interaction database [12]. In the virus target enrichment analysis, it was found that the 185 genes were significantly enriched in targets of the HIV-1 protein nef with a p value of 0.028. Within the 185 genes, there were 12 target genes of nef: HLA-C, IL2, PAK1, ICAM1, MMP9, MAP3K5, GFAP, CSF1R, TNF, IRF2, ELK1 and CBL. This result suggests that nef plays an important role in HIV-1 infection. In fact, it has been reported that in the early stages of the HIV-1 viral life cycle, the expression of nef promotes two major processes of HIV infection: T-cell activation and the establishment of a persistent state of infection [27]. It was observed in Sydney that the patients infected with nef-deleted virus take more time to progress to AIDS [28]. This clinical observation confirmed that nef plays an important role in HIV-1 infection. Therapies that affect the human protein targets of nef may weaken the virulence and infectivity of HIV-1 and increase HIV-1 resistance.

### Network analysis of virus-host interaction

To analyze virus-host interactions, we integrated the HIV-1, human protein interaction network and the STRING human protein interaction network. We calculated the modified betweenness, which is defined in the Methods section, and identified proteins that are important for communication and potentially transmit information from one virus-targeted protein to another. Twenty-nine genes out of the 185 HIV-1 resistance genes were infection information exchangers. For instance, CBL was on 19,859 of the shortest paths between virus-targeted proteins, and IL2 controls 12,939 virus target communication pathways. These proteins are important for exchanging information between virus-targeted proteins and coordinating virus infection. **Table S4** gives the 29 infection information exchanger genes from the 185 genes.

## Discussion

Based on the effective integration between the human protein interaction network and the STRING human protein interaction network, 29 infection information exchanger and HIV-1 resistance genes (see **Table S4**) were identified. The infection information exchanger genes are located on the shortest paths between virus-targeted proteins, which suggest that they play essential roles in the coordination of virus infection. The concept of infection information exchanger genes is useful for revealing key players in virus infection and for providing candidates for drug target screening. It can be easily applied to other virus-related studies, such as hepatitis B virus (HBV), hepatitis C virus (HCV) [29], and influenza A (H1N1) virus [10].

Within the 29 genes identified as infection information exchanger and HIV-1 resistance genes, some have been well studied. CBL (murine Cas-Br-M) was first identified as part of a transforming retrovirus that induces mouse pre-B and pro-B cell lymphomas [30]. CBL positively regulates receptor protein-tyrosine kinase ubiquitination as an adaptor dependent upon its variant SH2 and RING finger domains [31]. HIV nef modifies T cell signaling by enhancing CBL phosphorylation in the absence of T cell receptor engagement and co-stimulation [32]. Nef-mediated lipid raft exclusion inhibits CBL activity, which positively regulates signaling in T cells [33]. In our study, CBL was on 19,859 of the shortest paths, which suggests that it is important for HIV infection. The second gene on the list of 29 genes, IL2, is a secreted cytokine that regulates CD4+ T cell production and survival [34]. IL2 is also known to increase CD4 cell counts in HIV-infected patients [35]. Here, IL2 was implicated in the control of 12,939 virus target communication paths, contributing to the hypothesis that IL2 is involved in HIV entry. The third gene on the list of 29 genes, ABL1 (c-abl oncogene 1, non-receptor tyrosine kinase), encodes a tyrosine kinase involved in cell differentiation, cell division, cell adhesion, and stress responses [36], [37], [38]. ABL1 is negatively regulated by its SH3 domain, and deletion of the SH3 domain changes ABL1 into an oncogene [37]. The DNA-binding activity of ABL1 is regulated by CDC2-mediated phosphorylation [39]. ABL1 was on 7516 virus target communication paths in this study. This is the first evidence of a link between ABL1 and HIV infection.

We also found many other factors that would potentially be target genes in HIV infection, such as mitochondrial ribosomal protein S12 (MRPS12), nuclear factor erythroid-derived 2 (NFE2), mitogen-activated protein kinase 7 (MAPK7), CASP8 and FADD-like apoptosis regulator (CFLAR), and glutathione S-transferase alpha 4 (GSTA4). The identification of the targets cannot predict whether increasing or decreasing its function will generate resistance to HIV. Further investigation of these proteins during HIV infection is needed.

To summarize, we identified 185 HIV-1 resistance genes that discriminate between HIV-1-resistant samples and HIV-1-susceptible samples. The prediction accuracy was 85.2% evaluated by Leave-One-Out Cross-Validation (LOOCV). The virus target enrichment of the 185 genes suggests that the HIV-1 protein nef may play an important role in HIV-1 infection. A novel method for the analysis of virus-host interactions was proposed, and modified betweenness was used to measure the information exchange between virus-targeted proteins. Twenty-nine genes out of the 185 HIV-1 resistance genes were infection information exchangers, which were located on the shortest paths between virus-targeted proteins and may disrupt the communication between virus targets. These proteins are important for the coordination of virus infection, and therapies that affect the infection information exchanger genes may disrupt communication between virus-targeted proteins and HIV-1 infection. They are potential novel drug targets for AIDS therapy or prevention and may be important for understanding the mechanism of HIV-1 infection.

## Supporting Information

Table S1 **The HIV-1, human protein interactions from (** http://www.ncbi.nlm.nih.gov/RefSeq/HIVInteractions/ **).**
(XLS)

Table S2 **The 185 genes selected by mRMR and IFS.**
(XLS)

Table S3 **The Gene Ontology enrichment of the 185 genes using GATHER (** http://gather.genome.duke.edu/ **).**
(XLS)

Table S4 **Descriptions of 29 infection information exchanger gene identified from the 185 HIV-1 resistance genes.**
(PDF)
