# Supplementary material for: Computational Analysis of HIV-1 Resistance Based on Gene Expression Profiles and the Virus-Host Interaction Network
Source: PLoS One. 2011 Mar 4;6(3):e17291. doi: 10.1371/journal.pone.0017291 (PMC3048858; doi:10.1371/journal.pone.0017291)
Supplement: Table S4 — Descriptions of 29 infection information exchanger gene identified from the 185 HIV-1 resistance genes. (PDF) [file pone.0017291.s004.pdf]

Table S4 - Descriptions of 29 infection information exchanger gene identified from the 185 HIV-1 resistance genes

Gene Symbol	Target protein	Number of shortest paths between virus-targeted proteins	Description
CBL	Yes	19859	CBL is an oncogene that was first identified as part of a transforming retrovirus [1].
IL2	Yes	12939	IL2 regulates CD4+ T cell production and survival [2].
ABL1	No	7516	ABL1 is a proto-oncogene that encodes a cytoplasmic and nuclear protein tyrosine kinase [3].
PTEN	Yes	3942	Tat-dependent activation of the Egr1-PTEN-FOXO3a pathway provides a mechanism for HIV-1-associated CD4+ T cell death [4].
PAK1	Yes	3445	RAC1 to PAK1 and PI3K to PAK1 activation pathways are essential for nef-mediated pathogenesis of HIV [5].
GTF2B	Yes	3061	GTF2B interacts with HIV-1 Vpr [6].
TIMP1	Yes	2626	HIV infection increases TIMP1 levels [7].
SRF	No	1821	HIV-1 Tat is phosphorylated by serum response factor (SRF) [8].
TJP1	No	1821	HIV-1 gp120 proteins alter tight junction protein (TJP1) expression [9].
MRPS12	No	1814	Mitochondrial ribosomal protein S12 (MRPS12).
SOD2	Yes	1801	HIV-1 Tat regulates the SOD2 basal promoter by altering SP1/SP3 binding activity [10].
ICAM1	Yes	1528	HIV-1 Tat up-regulates expression of the ICAM1 and VCAM1 genes [11].
MAP3K5	Yes	1085	HIV-1 Nef inhibits MAP3K5 activity [12].
GHR	No	911	HIV gp120 inhibits GHR in the somatotropic axis during the pathogenesis of AIDS wasting [13].
RPS6KA5	No	911	Ribosomal protein S6 kinase, 90 kDa, polypeptide 5 (RPS6KA5).
NFE2	No	905	Nuclear factor erythroid-derived 2 (NFE2).
ITGA3	Yes	902	Integrin, alpha 3; antigen CD49C, alpha 3 subunit of VLA-3 receptor (ITGA3).
HGF	No	902	Overexpression of the HGF/c-Met complex is strongly correlated with oncogenic HIV infection [14].
PRKAR1A	Yes	779	PRKAR1A phosphorylates HIV Nef [15].
CD9	Yes	646	HIV-1 assembly into intracellular plasma membrane is dependent on Tetraspanins CD9 [16].
CDK6	No	453	HIV transactivator TAT binds to the

			CDK6-activating kinase [17].
MAPK7	No	138	Mitogen-activated protein kinase 7 (MAPK7).
CDC25A	No	85	HIV Vpr binds to CDC25 and inhibits CDC25 phosphatase activity [18].
SOD1	Yes	17	Tat decreased endogenous cellular, but not transduced, SOD1 [19].
PRKAR2B	Yes	16	PRKAR2B interacts with Tat [20].
MAFG	No	14	v-maf musculoaponeurotic fibrosarcoma oncogene homolog G (MAFG).
ADK	No	9	ADK is an HIV-1 integrase inhibitor [21].
CFLAR	No	5	CASP8 and FADD-like apoptosis regulator (CFLAR).
GSTA4	No	3	Glutathione S-transferase alpha 4 (GSTA4).

Reference

1. Langdon WY, Heath KG, Blake TJ (1992) The localization of the products of the c-cbl and v-cbl oncogenes during mitosis and transformation. Curr Top Microbiol Immunol 182: 467-474.

2. Kelly E, Won A, Refaeli Y, Van Parijs L (2002) IL-2 and related cytokines can promote T cell survival by activating AKT. J Immunol 168: 597-603.

3. Burton EA, Pendergast AM, Aballay A (2006) The Caenorhabditis elegans ABL-1 tyrosine kinase is required for Shigella flexneri pathogenesis. Appl Environ Microbiol 72: 5043-5051.

4. Cook JA, August A, Henderson AJ (2002) Recruitment of phosphatidylinositol 3-kinase to CD28 inhibits HIV transcription by a Tat-dependent mechanism. J Immunol 169: 254-260.

5. Renkema GH, Manninen A, Mann DA, Harris M, Saksela K (1999) Identification of the Nef-associated kinase as p21-activated kinase 2. Curr Biol 9: 1407-1410.

6. Agostini I, Navarro JM, Rey F, Bouhamdan M, Spire B, et al. (1996) The human immunodeficiency virus type 1 Vpr transactivator: cooperation with promoter-bound activator domains and binding to TFIIB. J Mol Biol 261: 599-606.

7. Leveque T, Le Pavec G, Boutet A, Tardieu M, Dormont D, et al. (2004) Differential regulation of gelatinase A and B and TIMP-1 and -2 by TNFalpha and HIV virions in astrocytes. Microbes Infect 6: 157-163.

8. Kim MS, Merlo X, Wilson C, Lough J (2006) Co-activation of atrial natriuretic factor promoter by Tip60 and serum response factor. J Biol Chem 281: 15082-15089.

9. Kanmogne GD, Primeaux C, Grammas P (2005) HIV-1 gp120 proteins alter tight junction protein expression and brain endothelial cell permeability: implications for the pathogenesis of HIV-associated dementia. J Neuropathol Exp Neurol 64: 498-505.
10. Marecki JC, Cota-Gomez A, Vaitaitis GM, Honda JR, Porntadavity S, et al. (2004) HIV-1 Tat regulates the SOD2 basal promoter by altering Sp1/Sp3 binding activity. Free Radic Biol Med 37: 869-880.
11. Song HY, Ryu J, Ju SM, Park LJ, Lee JA, et al. (2007) Extracellular HIV-1 Tat enhances monocyte adhesion by up-regulation of ICAM-1 and VCAM-1 gene expression via ROS-dependent NF-kappaB activation in astrocytes. Exp Mol Med 39: 27-37.
12. Geleziunas R, Xu W, Takeda K, Ichijo H, Greene WC (2001) HIV-1 Nef inhibits ASK1-dependent death signalling providing a potential mechanism for protecting the infected host cell. Nature 410: 834-838.
13. Mulroney SE, McDonnell KJ, Pert CB, Ruff MR, Resch Z, et al. (1998) HIV gp120 inhibits the somatotropic axis: a possible GH-releasing hormone receptor mechanism for the pathogenesis of AIDS wasting. Proc Natl Acad Sci U S A 95: 1927-1932.
14. Walker F, Kermorgant S, Darai E, Madelenat P, Cremieux AC, et al. (2003) Hepatocyte growth factor and c-Met in cervical intraepithelial neoplasia: overexpression of proteins associated with oncogenic human papillomavirus and human immunodeficiency virus. Clin Cancer Res 9: 273-284.
15. Li PL, Wang T, Buckley KA, Chenine AL, Popov S, et al. (2005) Phosphorylation of HIV Nef by cAMP-dependent protein kinase. Virology 331: 367-374.
16. Gordon-Alonso M, Yanez-Mo M, Barreiro O, Alvarez S, Munoz-Fernandez MA, et al. (2006) Tetraspanins CD9 and CD81 modulate HIV-1-induced membrane fusion. J Immunol 177: 5129-5137.
17. Ezhevsky SA, Nagahara H, Vocero-Akbani AM, Gius DR, Wei MC, et al. (1997) Hypo-phosphorylation of the retinoblastoma protein (pRb) by cyclin D:Cdk4/6 complexes results in active pRb. Proc Natl Acad Sci U S A 94: 10699-10704.
18. Huard S, Elder RT, Liang D, Li G, Zhao RY (2008) Human immunodeficiency virus type 1 Vpr induces cell cycle G2 arrest through Srk1/MK2-mediated phosphorylation of Cdc25. J Virol 82: 2904-2917.
19. Agrawal L, Louboutin JP, Strayer DS (2007) Preventing HIV-1 Tat-induced neuronal apoptosis using antioxidant enzymes: mechanistic and therapeutic implications. Virology 363: 462-472.
20. Zidovetzki R, Wang JL, Chen P, Jeyaseelan R, Hofman F (1998) Human immunodeficiency virus Tat protein induces interleukin 6 mRNA expression

in human brain endothelial cells via protein kinase C- and cAMP-dependent protein kinase pathways. AIDS Res Hum Retroviruses 14: 825-833.

21. Zhang X, Pais GC, Svarovskaia ES, Marchand C, Johnson AA, et al. (2003) Azido-containing aryl beta-diketo acid HIV-1 integrase inhibitors. Bioorg Med Chem Lett 13: 1215-1219.
